# SkinSensDB: a curated database for skin sensitization assays

**DOI:** 10.1186/s13321-017-0194-2

**Published:** 2017-01-31

**Authors:** Chia-Chi Wang, Ying-Chi Lin, Shan-Shan Wang, Chieh Shih, Yi-Hui Lin, Chun-Wei Tung

**Affiliations:** 1 School of Pharmacy, Kaohsiung Medical University, 100 Shih-Chuan 1st Road, Kaohsiung, 80708 Taiwan; 2 PhD Program in Toxicology, Kaohsiung Medical University, Kaohsiung, 80708 Taiwan; 3 National Institute of Environmental Health Sciences, National Health Research Institutes, Miaoli County, 35053 Taiwan; 4 Institute of Environmental Engineering, National Sun Yat-sen University, Kaohsiung, 80424 Taiwan; 5 Research Center for Environmental Medicine, Kaohsiung Medical University, Kaohsiung, 80708 Taiwan

## Abstract

Skin sensitization is an important toxicological endpoint for chemical hazard determination and safety assessment. Prediction of chemical skin sensitizer had traditionally relied on data from rodent models. The development of the adverse outcome pathway (AOP) and associated alternative in vitro assays have reshaped the assessment of skin sensitizers. The integration of multiple assays as key events in the AOP has been shown to have improved prediction performance. Current computational models to predict skin sensitization mainly based on in vivo assays without incorporating alternative in vitro assays. However, there are few freely available databases integrating both the in vivo and the in vitro skin sensitization assays for development of AOP-based skin sensitization prediction models. To facilitate the development of AOP-based prediction models, a skin sensitization database named SkinSensDB has been constructed by curating data from published AOP-related assays. In addition to providing datasets for developing computational models, SkinSensDB is equipped with browsing and search tools which enable the assessment of new compounds for their skin sensitization potentials based on data from structurally similar compounds. SkinSensDB is publicly available at http://cwtung.kmu.edu.tw/skinsensdb.

## Background

Skin sensitization associated with allergic contact dermatitis (ACD) is the second most common occupational illness accounting for 10–15% of all occupational disease worldwide [1]. The disease not only impairs the quality of life for the patients but also results in high costs in healthcare systems and economy [2]. Skin sensitization is thereby an important toxicological endpoint in chemical safety assessment and a focus in regulatory decision making. Chemical sensitizers, which may be detergents, preservatives, or fragrances in household and personal care products or active ingredients, impurities from synthetic process and industrial materials in the pharmaceutical products, act as haptens binding to protein molecules. These chemically modified proteins may trigger T cell-mediated immune reactions and lead to ACD [3].

Traditionally, guinea pig maximization test (GPMT) and Buehler assay (BA) are utilized as predictive animal models for the identification of skin sensitizers and are widely accepted by regulatory authorities due to their reliable detection of potential human contact allergens. Nevertheless, these protocols are relatively long and complex with some limitations [4]. The murine local lymph node assay (LLNA) has been established as an alternative animal model to traditional guinea pig methods to provide important animal welfare benefits and has been successfully validated and incorporated into a regulatory guideline described as Organization for Economic Cooperation and Development (OECD) Test Guideline Number 429 [5]. The assay measures the proliferation of lymphocytes during the induction phase of skin sensitization and provides EC3 values (effective concentration for a stimulation index of threefold in lymphocyte proliferation compared to vehicle controls) to make comparisons of the relative potency of different chemical sensitizers. The animal tests have been recently banned for cosmetic ingredients in 2013 by the European Union. There is a strong need for the development of alternative testing methods for identifying skin sensitizers [6, 7].

Many quantitative structure–activity relationship (QSAR) methods have been proposed to predict skin sensitizers based on descriptors of chemical structures [8–18]. All of the QSAR models were developed for a single type of sensitization endpoints mostly from either LLNA or GPMT. Although reasonably good prediction performance was obtained from the QSAR models, the prediction gave no insights into the detailed mechanism of actions. Recently, skin sensitization has been formulated as an adverse outcome pathway (AOP) to address the complex nature of sensitization [19]. Four key events including protein binding, keratinocyte activation, dendritic cell activation and T cell activation were clearly defined. Several alternative methods have been developed according to the key events such as Direct Peptide Reactivity Assay (DPRA) and Peroxidase Peptide Reactivity Assay (PPRA) [20, 21] for quantification of chemical peptide reactivity, KeratinoSens [22, 23] and LuSens [24] for keratinocyte activation by determining the activation of antioxidant response element (ARE) reporter genes, and h-CLAT [25, 26] for activation of dendritic cells by measuring the increased level of CD54 and CD86 maturation markers, respectively. The integration of the key events of the AOP for predicting skin sensitizers showed improved prediction performance over single models [27–30]. In addition, the data from individual steps of the sensitization progress can be interpreted and evaluated by experts for hazard determination and risk assessment.

To facilitate the development of AOP-based computational prediction methods, a novel curated database named SkinSensDB was constructed by manual curation of published literature. A total of 710 unique chemicals were curated into SkinSensDB with corresponding reactivities of 2078, 467, 1323 and 1060 assay values for peptide reactivity, keratinocyte activation, dendritic cell activation, and T-cell activation, respectively. Search tools have been implemented with exact, similarity, and substructure search functionality. The SkinSensDB is expected to be a useful database supporting the development of AOP-based computational models for predicting skin sensitizers.

## Construction and content

The SkinSensDB database was implemented using MongoDB version 3.0.7. The web interface and search functions were implemented using PHP, Python, HTML and JavaScript programming languages and frameworks of AngularJS version 1.4.6 (https://angularjs.org/) and Angular Material version 1.1.1 (https://material.angularjs.org/latest/). The SkinSensDB website is publicly available at http://cwtung.kmu.edu.tw/skinsensdb.

### Database content

SkinSensDB database consists of chemical information and four types of well-developed assays associated with the four events of the AOP for skin sensitization reported by OECD [19]. For each chemical, its basic structure and physicochemical property information was extracted from PubChem Compound database using PUG REST functions from PubChem [31]. Chemical-related information provided at SkinSensDB included the CAS number, IUPAC name, INCHI, INCHIKEY, formula, SMILES, hydrogen-bond acceptor, hydrogen-bond donor, molecular weight and topological polar surface area (TPSA). In addition, external links to PubChem database and SDF files for 2D and 3D structures were available for further exploration of chemical-specific information and development of QSAR models, respectively.

For protein binding ability of chemicals, results of peptide reactivity assays of DPRA/PPRA were collected. For each chemical, its corresponding curated information included source literature, assay types, markers, peptide concentrations, chemical concentrations, the addition of horseradish peroxidase (HRP) enzymes and the percentage of peptide depletion with standard deviations. Peptide depletion assays with and without enzymatic activation using HRP enzymes are so called PPRA and DPRA, respectively. The keratinocyte activation ability of chemicals was represented by data from KeratinoSens and LuSens assays. Curated information for keratinocyte activation included the maximum fold induction of luciferase activity (Imax), concentrations for the *n*-fold induction of luciferase (EC1.5, EC2, and EC3) and cytotoxicity (IC50). For the activation potential of dendritic cells, information related to h-CLAT was curated including CD54 and CD86 markers, the induction criteria for the markers (EC type), the effective concentrations for the markers (EC), relative fluorescence intensity (RFI), and the concentrations that produced 75% cell viability (CV75). The LLNA assays representing the T-cell activation ability of chemicals were collected from NICEATM LLNA database [32]. LLNA-associated data consisted of vehicles, effective concentration for threefold induction of draining lymph node cell proliferation, the result of LLNA, evaluated concentrations and corresponding stimulation index (SI).

## Utility and discussion

**Fig. 1:**
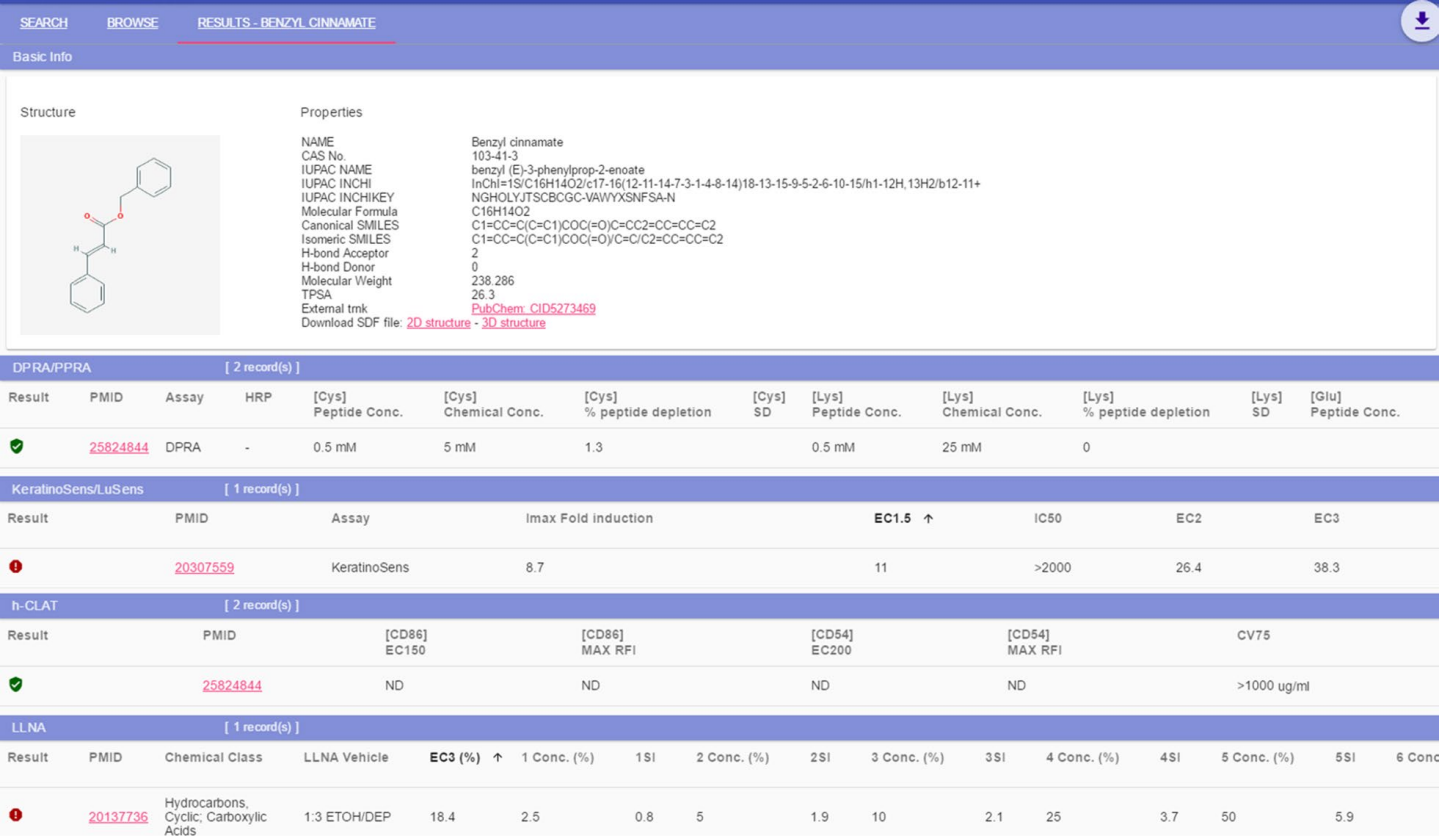
An illustrated record of SkinSensDB. For simplicity, *only one row* for each assay is included in this figure. The section of Basic Information shows the chemical structure and physicochemical properties with links to PubChem database and structure files in SDF format. Four sections comprise assay results corresponding to the four key events of adverse outcome pathway for skin sensitization

SkinSensDB is a curated database for AOP-associated skin sensitization assays aiming to provide an easily accessible resource for the development of AOP-based computational models. Currently, there are 710 unique chemicals with 4928 assay values of AOP-related skin sensitization assays from the literature. A typical record of SkinSensDB is shown in Fig. 1 where basic information of chemicals and associated skin sensitization assays can be found. The full record can be exported as an Excel file via the download button. Both the 2D and 3D structure files are downloadable for each chemical. With the integration of chemical structure and physicochemical property information, it is expected to be useful for developing AOP-based QSAR models. Users who wish to contribute their data to this database can submit their data using an Excel file provided at SkinSensDB.

For the exploration of chemicals and corresponding skin sensitization assays, a browse tool providing searchable summarized information of the four assays has been implemented. The criteria for defining the summarized information were shown in Table 1. The data table can be easily browsed by sorting and filtering on specific columns. Functions for batch queries and exporting the summarized information were also implemented.

**Table 1:** The classification criteria of positive and negative responses to a chemical

Assay type	Criteria	Classification
DPRA/PPRA	Peptide depletion ≤ 6.38%	Negative
	Peptide depletion > 6.38%	Positive
KeratinoSens/LuSens	EC1.5 ≥ 1000 µM	Negative
	EC1.5 < 1000 µM	Positive
h-CLAT	Neither CD86 EC150 nor CD54 EC200 was determined	Negative
	CD86 EC150 ≤ CV75 or CD54 EC200 ≤ CV75	Positive
LLNA	SI < 3	Negative
	SI ≥ 3	Positive

*EC* effective concentration, *CV75* 75% cell viability, *SI* stimulation index

**Fig. 2:**
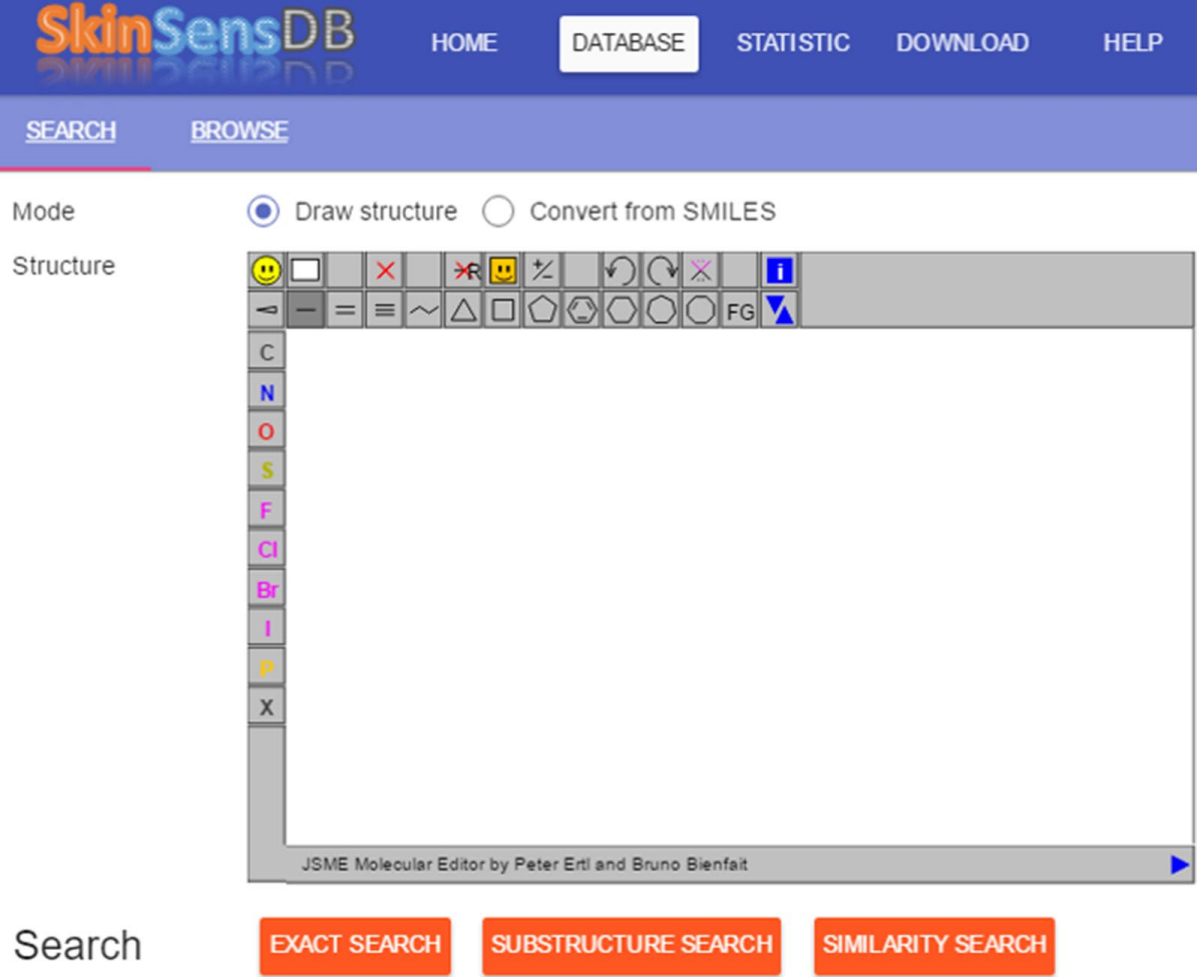
The search functions. Users can search the SkinSensDB database using functions of exact, substructure and similarity searches with a user-supplied chemical structure by either drawing a chemical structure or converting from a SMILES string

**Fig. 3:**
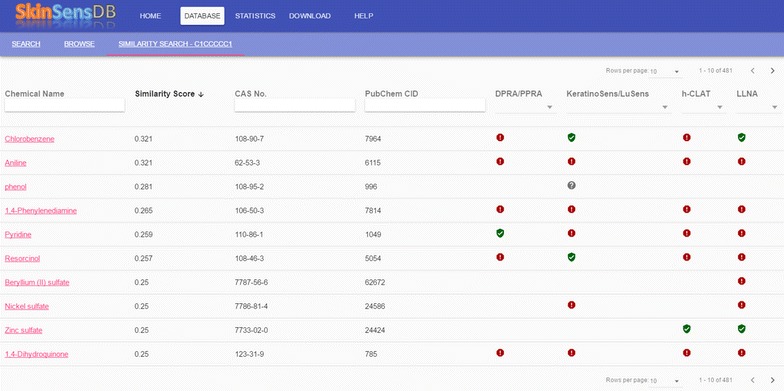
An illustrated example of the similarity search in SkinSensDB. A similarity score between query and target chemicals is available and sortable in the second column. All the other columns are the same as the browse tool in SkinSensDB consisting of name, CAS number, PubChem CID, summarized results for the four key events

Based on the assumption that structurally similar chemicals could have similar bioactivities, three types of tools for exact, similarity and substructure searches have been implemented to facilitate the search of structurally similar chemicals. Figure 2 shows the user interface of search functions. For the input of chemical structure for search, users can either draw a structure in the JSME [33] editor or enter a SMILES text that can be automatically converted into chemical structures. The search functions were implemented based on RDKit [34], an open-source cheminformatics library. The similarity search was based on Tanimoto similarity between topological fingerprints of two chemicals. In addition to the summarized information, the similarity score between 0 and 1 will be available in the search results as shown in Fig. 3.

In addition, a file summarizing essential information for developing QSAR models is available at the SkinSensDB website. The summarized file consists of fields of the chemical name, CAS number, PubChem CID, the highest peptide depletion of DPRA/PPRA, the lowest EC1.5 value of KeratinoSens/LuSens, the lowest EC and CV75 values of h-CLAT, and the EC3 value of LLNA for all chemicals in SkinSensDB. The structure files can be easily downloaded from PubChem database using the CIDs and transformed into descriptors using softwares such as PaDEL-Descriptor [35]. QSAR models can be subsequently constructed to study the relationship between structure descriptors and response values from assays of four key events.

## Conclusions

SkinSensDB is a web-based resource providing useful information of chemical structures, physicochemical properties and experimental data from both alternative in vitro and in vivo skin sensitization assays. Browse and search tools were implemented to facilitate the exploration of skin sensitization data. The integration of chemical structures, physicochemical properties, and experimental results from these AOP-related assays could be helpful for the development of an AOP-based prediction system integrating all models corresponding to the four major events.
